# Supplementary material for: Predicting visual function by interpreting a neuronal wiring diagram
Source: Nature. 2024 Oct 2;634(8032):113–23. doi: 10.1038/s41586-024-07953-5 (PMC11446822; doi:10.1038/s41586-024-07953-5)
Supplement: Supplementary file 6 — CRF and ERF predictions for individual TmY4 and TmY9 cells. Analogous to Supplementary Data 3, but for TmY target types. Shown are the top four monosynaptic pathways, the strongest pathway passing through each of the top ten intermediary types (ranking from Extended Data Fig. 7), and the trisynaptic pathway Tm1–TmY–Dm3–TmY (see the section entitled Prediction of spatial normalization). [file 41586_2024_7953_MOESM6_ESM.zip › DataS4/TmY9qΓèÑ/720575940631183391.pdf]

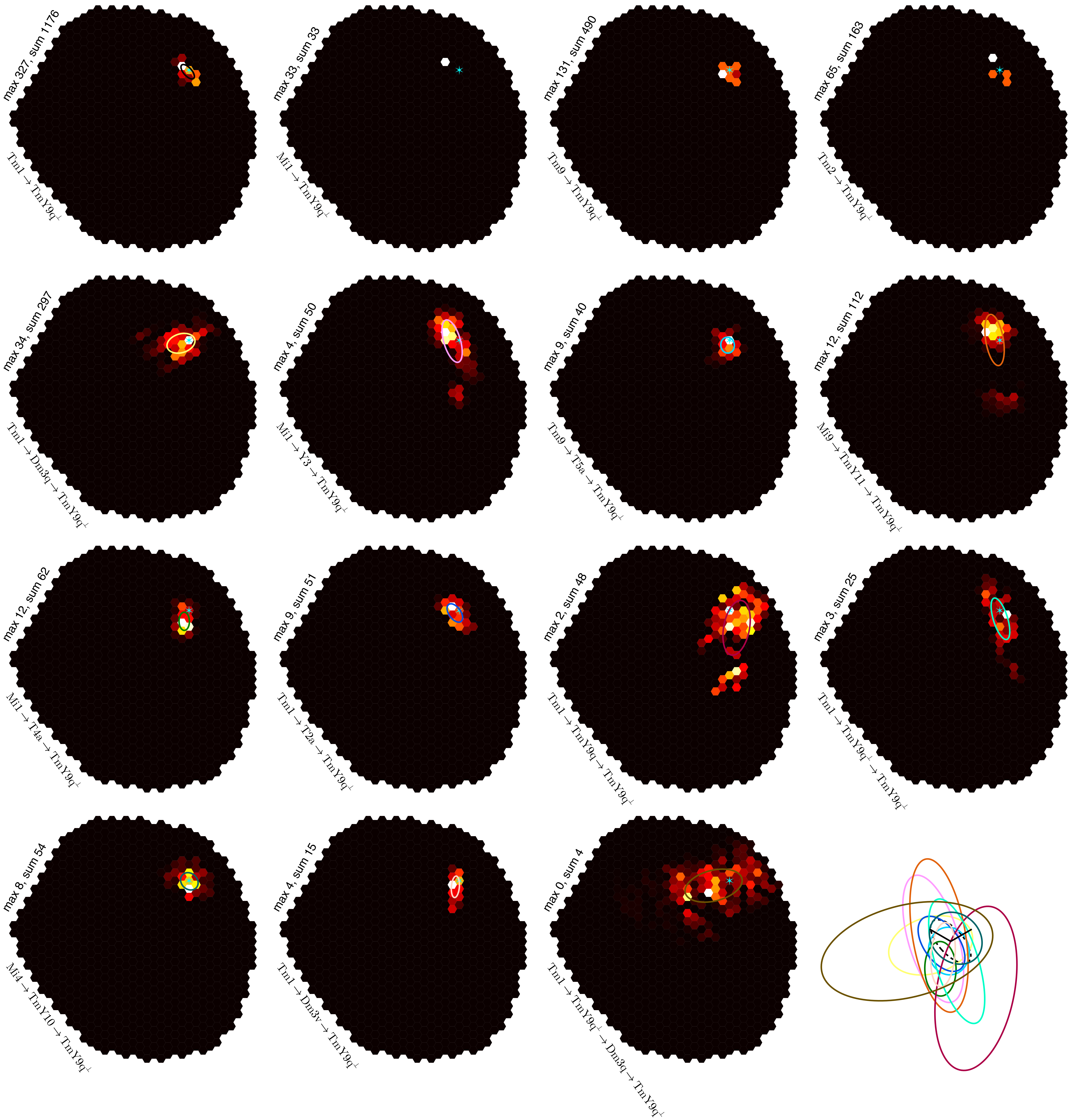
max 327, sum 1176
Tm1 → TmY9q⊥
max 33, sum 33
Mi1 → TmY9q⊥
max 131, sum 490
Tm9 → TmY9q⊥
max 65, sum 163
Tm2 → TmY9q⊥
max 34, sum 297
Tm1 → Dm3q → TmY9q⊥
max 4, sum 50
Mi1 → Y3 → TmY9q⊥
max 9, sum 40
Tm9 → T5a → TmY9q⊥
max 12, sum 112
Mi9 → TmY11 → TmY9q⊥
max 12, sum 62
Mi1 → T4a → TmY9q⊥
max 9, sum 51
Tm1 → T2a → TmY9q⊥
max 2, sum 48
Tm1 → TmY9q⊥ → TmY9q⊥
max 3, sum 25
Tm1 → TmY9q⊥ → TmY9q⊥
max 8, sum 54
Mi4 → TmY10 → TmY9q⊥
max 4, sum 15
Tm1 → Dm3v → TmY9q⊥
max 0, sum 4
Tm1 → TmY9q⊥ → Dm3q → TmY9q⊥
